# Immune cell and cytokine profiles in a murine *Aspergillus fumigatus* extract sensitization model

**DOI:** 10.7555/JBR.39.20250479

**Published:** 2026-09-28

**Authors:** Long Liang, Ying Luo, Yuqiang Pei, Yongchang Sun

**Affiliations:** Department of Respiratory and Critical Care Medicine, Peking University Third Hospital, Beijing 100191, China

Dear Editor,

Fungal allergen sensitization is a clinically important gateway event in difficult-to-treat asthma, and *Aspergillus fumigatus* is among the most consequential aeroallergens associated with exacerbation risk and morbidity^[1]^. Sensitization programs T helper (Th) cell lineages, mobilizes innate effectors, and establishes downstream airway pathology. Traditionally, this is described as type 2 inflammation (driven by type 2 cytokines including interleukin-4 [IL-4], IL-5, and IL-13), with accumulating evidence that Th9/IL-9 contributes to mucus production, mast cell activation, and airway hyperresponsiveness^[2–3]^. However, three barriers have limited mechanistic resolution during the sensitization window. First, inherent heterogeneity and lot-to-lot variability in crude, natural-source allergen extracts hinder standardization across experiments, thereby reducing reproducibility and obscuring causal inference regarding the earliest immunologic events following sensitization^[4]^. Second, most studies interrogate only one layer (serology, leukocyte phenotypes, or cytokine transcription), making it difficult to link antigen exposure to T helper cell programming and subsequent effector mobilization within the same animals and time frame. Third, studies that simultaneously profile the lung and peripheral blood have been scarce, even though granulocyte dynamics (*e.g*., eosinophil release into the blood versus airway infiltration) and dendritic cell migration are significant as biomarkers for allergic disease^[5]^.

To address these gaps, we established a standardized murine sensitization model using inactivated *A. fumigatus* extract (AFE; Stallergenes Greer, Lenoir, NC, USA; Cat. #XPM3D3A4), a protein-enriched extract derived from *A. fumigatus* culture filtrates and containing multiple soluble allergens. Female C57BL/6N mice aged 4–6 weeks were obtained from the Department of Animal Laboratory Science of Peking University. After a one-week acclimatization period, the mice were randomly divided into two groups, with five mice in each group (control and AFE-model groups). To establish the *A. fumigatus* sensitization model, mice received subcutaneous injections of 100 µL AFE (1 mg/mL) on days 1 and 8, followed by intranasal administration of 25 µL AFE once per week for three consecutive weeks under isoflurane anesthesia (***Fig. 1A***). To determine whether early AFE sensitization engages the canonical signaling axes that characterize type 2 (T2)-high and fungus-associated asthma, we selected readouts that capture IgE production, T helper cell polarization, granulocyte recruitment, and key cytokine/chemokine signals. We measured (i) total IgE (t-IgE) and *A. fumigatus*-specific IgE (Af-IgE) in serum by enzyme-linked immunosorbent assay; (ii) lung and blood immune cell populations by multiparameter flow cytometry, including Th1/Th2/Th9/Th17/Treg CD4^+^ T cell subsets, B cells, dendritic cells (DCs), alveolar and interstitial macrophages, eosinophils, neutrophils, and monocytes; and (iii) lung cytokine/chemokine transcripts (IL-4, IL-5, IL-9, IL-13, CCL17, and Th1/Th22 markers) by real-time reverse transcription-qPCR (RT-qPCR), with reactions performed in technical triplicate. Data distributions were assessed for normality using the Shapiro–Wilk test. Comparisons between the two groups were performed using unpaired two-tailed Student's *t*-tests, with significance set at *P* < 0.05. Details of antibody panels, gating markers, and qPCR primers are provided in ***Supplementary Tables 1***–***3***. All procedures were approved by the Ethics Committee of Peking University Third Hospital (Approval No. SA2024664).

**Figure 1:**
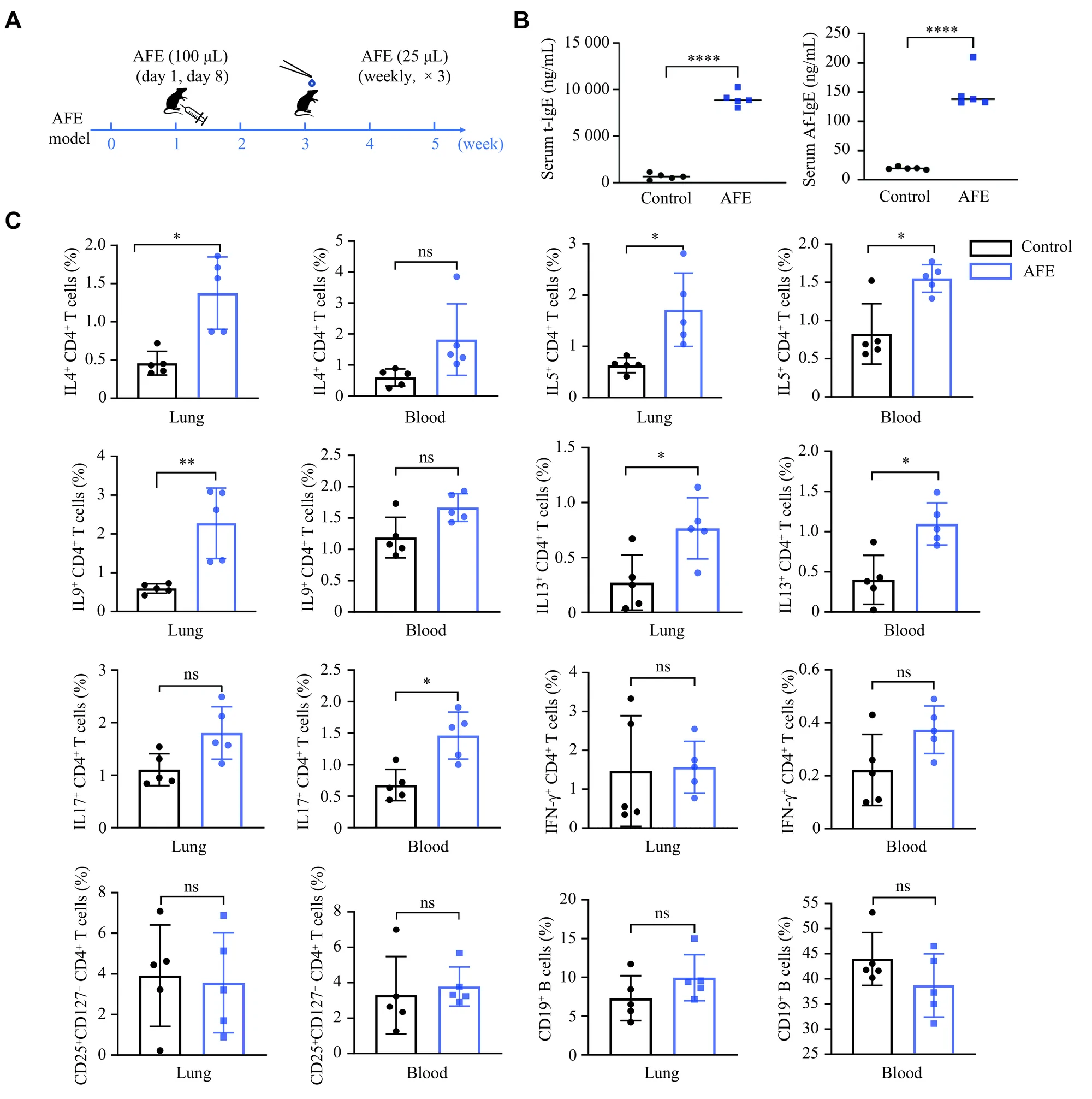
Establishment and immunological characterization of AFE-sensitized mouse model. A: Construction of a mouse model of sensitization by *Aspergillus fumigatus*. B: Detection of serum t-IgE and Af-IgE levels. C: CD4^+^ T cell subset alterations in lung and blood of AFE-sensitized mice. Flow cytometric analysis showing frequencies of CD4^+^ T cells expressing IL-4, IL-5, IL-9, IL-13, IL-17, and IFN-γ, as well as Treg cells and CD19^+^ B cells, in lung and blood of AFE-sensitized mice. Data are presented as mean ± standard deviation, with individual data points shown; *n* = 5 mice per group. Statistical comparisons between the control and AFE groups were performed using unpaired two-tailed Student's *t*-tests. ^*^P < 0.05, ^**^P < 0.01, and ^****^P < 0.0001. Abbreviations: AFE, *A. fumigatus* extract; Af-IgE, *A. fumigatus*-specific IgE; IFN-γ, interferon gamma; ns, not significant; t-IgE, total immunoglobulin E; Treg, regulatory T cell.

AFE sensitization generated a strong humoral signature of allergen-specific sensitization. Relative to control mice, AFE-exposed mice showed marked increases in circulating t-IgE (9021 *vs.* 683.7 ng/mL; *P* < 0.0001) and Af-IgE (151.4 *vs.* 20.08 ng/mL; *P* < 0.0001), confirming robust acquisition of fungus-specific reactivity early after sensitization (***Fig. 1B***). At the T cell level, AFE sensitization induced coordinated Th2/Th9 polarization in the lung. The proportions of IL-4^+^, IL-5^+^, IL-13^+^, and IL-9^+^ CD4^+^ T cells were all increased in the lungs of AFE-sensitized mice compared with those of the controls (IL-4^+^ 1.38% *vs.* 0.46%, *P* = 0.0033; IL-5^+^ 1.64% *vs.* 0.63%, *P* = 0.0267; IL-13^+^ 0.75% *vs.* 0.27%, *P* = 0.0394; IL-9^+^ 2.27% *vs.* 0.61%, *P* = 0.0089; ***Fig. 1C*** and ***Supplementary Fig. 1***). In peripheral blood, IL-5^+^ and IL-13^+^ CD4^+^ T cells were similarly elevated (1.53% *vs.* 0.85%, *P* = 0.0332; 1.07% *vs.* 0.41%, *P* = 0.0278), whereas IL-4^+^ and IL-9^+^ CD4^+^ T cells showed non-significant trends toward higher frequencies (1.94% *vs.* 0.56%, *P* = 0.0829; 1.71% *vs.* 1.22%, *P* = 0.0655). IL-17^+^ CD4^+^ T cells increased significantly in the blood (1.42% *vs*. 0.72%, *P* = 0.0300) and showed a positive trend in the lung (1.85% *vs*. 1.15%, *P* = 0.0759). This pattern suggests that Th17 activation accompanies, but does not dominate over, the Th2/Th9 response during the sensitization phase and may provide an additional axis linking fungal sensitization to downstream neutrophilic and epithelial responses. In contrast, Th1 (IFN-γ^+^; 1.57% *vs*. 1.41%; *P* = 0.8292), Tregs (CD25^+^CD127^−^; 3.56% *vs*. 3.92%; *P* = 0.8270), and B cells (CD19^+^; 10.09% *vs*. 7.52%; *P* = 0.3191) in the lung did not differ significantly between groups. Similar patterns were observed in peripheral blood. Together, these results identify a dominant Th2/Th9 program with a measurable Th17 component in the circulation, rather than a Th1-driven response within the sensitization window, while some circulating Th2/Th9 readouts (*e.g.*, IL-4^+^ and IL-9^+^ CD4^+^ T cells) should be interpreted as non-significant but suggestive trends at this time point.

AFE sensitization also reshaped myeloid and innate compartments in a way that supports antigen capture and T2 effector programming rather than acute neutrophilic inflammation. In the lung, alveolar macrophages (F4/80^+^CD11c^+^) were significantly increased after sensitization (3.89% *vs*. 1.25%; *P* = 0.03; ***Fig. 2A*** and ***Supplementary Fig. 2A***–***2C***), suggesting enhanced local phagocytic and regulatory activity in the airway space. Interstitial macrophages (F4/80^+^CD11b^+^CD11c^−^) and classically activated (M1, CD86^+^) or alternatively activated (M2, CD206^+^) macrophage subsets in the lung did not differ significantly from controls. In blood, the total macrophage pool (F4/80^+^CD11b^+^Ly6C^+^) remained stable, and M1-like cells were unchanged, but M2-like macrophages increased significantly (3.13% *vs.* 0.31%; *P* = 0.0473), suggesting early systemic conditioning toward T2-associated repair/tolerance phenotypes.

**Figure 2:**
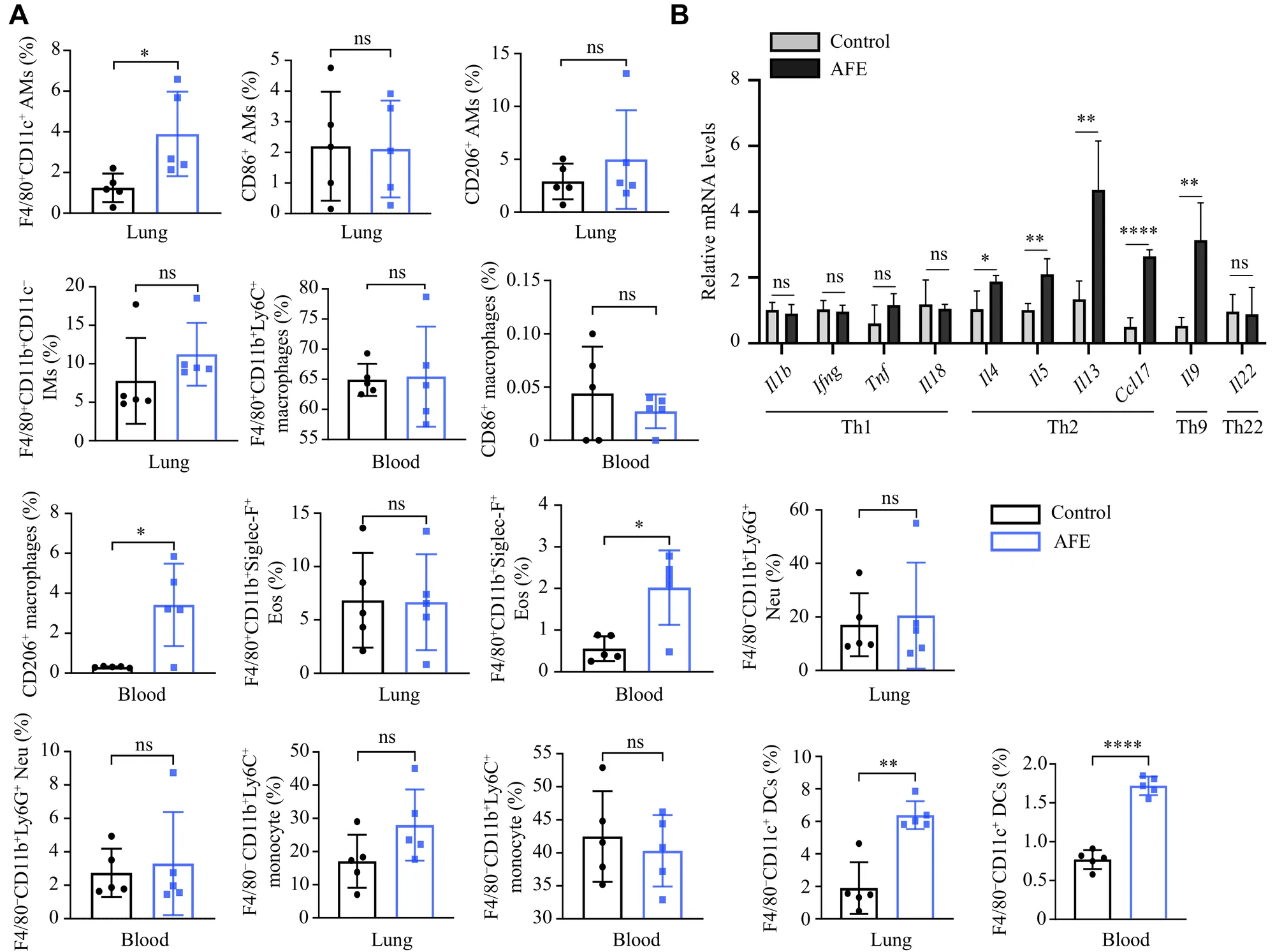
Proportions of immune cell subsets and cytokine profiles in the lung and blood of AFE-sensitized mice. A: Characterization of myeloid cell subsets in lung and blood of AFE-sensitized mice. This summarizes the ratios of major immune cell populations in the lung and blood, including AMs and their subsets, IMs, eosinophils, neutrophils, monocytes, and DCs. B: AFE sensitization induces a Th2/Th9-dominant cytokine profile in lung tissues. Data are presented as mean ± standard deviation, with individual data points shown; *n* = 5 mice per group. Statistical comparisons between the control and AFE groups were performed using unpaired two-tailed Student's *t*-tests. ^*^P < 0.05, ^**^P < 0.01, and ^****^P < 0.0001. Abbreviations: AMs, alveolar macrophages; DCs, dendritic cells; Eos, eosinophils; IMs, interstitial macrophages; M1/M2, M1-like/M2-like macrophages; Neu, neutrophils; ns, not significant.

We next examined granulocyte behavior using F4/80^+^CD11b^+^Siglec-F^+^ to define eosinophils. We found no significant change in eosinophil frequency in the lung at this time point (7.01% *vs.* 7.46%; *P* = 0.9025; ***Fig. 2A*** and ***Supplementary Fig. 2D***), but circulating eosinophils were significantly increased (1.96% *vs*. 0.59%; *P* = 0.0441). This discrepancy between lung and blood is consistent with the possibility that eosinophils are initially mobilized into the circulation before substantial accumulation in lung tissue; however, longitudinal studies are needed to establish the temporal sequence of eosinophil recruitment. Neutrophils (F4/80^−^CD11b^+^Ly6G^+^) and monocytes (F4/80^−^CD11b^+^Ly6C^+^) were unchanged in both compartments (***Fig. 2A*** and ***Supplementary Fig. 2E*** and ***2F***). Notably, DCs (F4/80^−^CD11c^+^) expanded in both the lung (6.38% *vs*. 2.00%; *P* = 0.0055) and blood (1.69% *vs*. 0.77%; *P* < 0.0001; ***Fig. 2A*** and ***Supplementary Fig. 2G***). This expansion of DCs may reflect enhanced antigen-presenting activity and is consistent with prior models in which airway DCs bridge innate sensing and adaptive Th2 polarization during allergic sensitization and asthma pathogenesis^[6–7]^.

Lung RT-qPCR corroborated this cellular pattern at the transcriptional level. Th2-associated cytokines *Il4*, *Il5*, *Il13*, Th2-recruiting chemokine *Ccl17*, and Th9 signature cytokine *Il9* were all significantly upregulated in lung tissues from AFE-sensitized mice (*P* = 0.0335, 0.0020, 0.0166, 0.0013, and 0.0190, respectively; ***Fig. 2B***). By contrast, transcripts linked to Th1 (*Il1b*, *Ifng*, *Tnf*, *Il18*) and Th22 (*Il22*) showed no significant changes. Thus, the cytokine/chemokine milieu in lung tissues mirrors the flow cytometry data and further supports a Th2/Th9-dominant, rather than Th1/Th22 sensitization profile. Although direct comparison between these fundamentally different disease models should be made cautiously, transcriptome-wide data from systemic *A. fumigatus* infection models have demonstrated predominant Th1/ Th17-type signatures in murine kidneys during invasive aspergillosis^[8]^. In contrast, our lung sensitization model shows a Th2/Th9-dominant signature with only an emerging Th17 component, underscoring how organ site (kidney *vs.* lung) and disease phase (invasive infection *vs.* allergic sensitization) shape the architecture of the host response.

Together, these findings outline an integrated portrait of early *A. fumigatus* sensitization. First, AFE exposure rapidly induced fungus-specific IgE and systemic T2 priming. Second, lung and circulating CD4^+^ T cells polarized toward Th2 and Th9, with concordant induction of IL-4/IL-5/IL-13/IL-9 and CCL17 transcripts in the lung. Third, DCs expanded in both the lung and blood, aligning with their role in antigen capture and Th2 instruction at the airway interface. Fourth, eosinophils were mobilized into the circulation while lung eosinophil frequency remained unchanged at this sensitization time point, consistent with a staged recruitment process. Finally, neutrophils and monocytes remained largely unchanged, arguing against an early neutrophil-dominant pattern. This pattern is consistent with previous models of *A. fumigatus*-induced pulmonary hypersensitivity, in which crude *A. fumigatus* antigens and allergens drive Th2-biased pulmonary hypersensitivity with high fungus-specific IgE, blood eosinophilia, and airway eosinophilic infiltration, and in which surfactant proteins A and D modulate the magnitude of these responses^[9]^. Our AFE sensitization model extends this work by mapping, in parallel, the associated Th9 and emerging Th17 signatures and by quantifying corresponding changes in blood versus lung compartments. Conceptually, these changes define a sensitization-phase network. In this network, DC amplification, Th2/Th9 polarization, and CCL17 induction cooperate to prepare the lung for T2-biased inflammation, whereas systemic eosinophil mobilization and emerging Th17 activity signal parallel peripheral engagement. It is also important to note that our protocol combines subcutaneous priming with intranasal boosting, so the resulting Th2/Th9-dominant response with an emerging Th17 component likely reflects the integration of systemic and mucosal antigen presentation; alternative sensitization and challenge routes (for example, purely intranasal or intratracheal allergen delivery) have been shown in other asthma models to skew the balance between Th2 and Th17 responses, and direct route-comparison studies will be needed to generalize our findings across exposure scenarios. These allergen-driven axes are also aligned with emerging evidence on traffic-related air pollution (TRAP). Birth-cohort studies link early-life TRAP exposure to increased childhood asthma incidence, and experimental models show that nitrogen dioxide (NO_2_) and related pollutants aggravate oxidative stress and allergic airway inflammation^[10–11]^. Together, these observations suggest that TRAP and fungal sensitization converge on overlapping T2-skewed and oxidative stress pathways.

Several limitations should be acknowledged, including the small sample size, assessment at a single time point, absence of direct measurements of airway hyperresponsiveness and histopathologic remodeling, and the inability of the present design to establish the temporal sequence or causal relationships among the observed immune changes. This platform provides a reproducible baseline for longitudinal studies and targeted interventions. Because we acquire serology, cellular phenotypes in both lung and blood, and lung transcriptional programs in the same animals and at the same time point, the model is well-suited for several applications. It can be used for (i) time-course mapping of eosinophil infiltration, airway hyperresponsiveness, and mucus remodeling; (ii) pathway-directed blockade of IL-4/IL-13, IL-5, IL-9, or CCL17 to test causal roles in sensitization; and (iii) translational biomarker work leveraging blood eosinophils, Th2/Th9 frequencies, or DC expansion as tractable readouts for T2-high airway disease. These axes are central to biologic therapy development in severe asthma and related phenotypes^[12–13]^. In future studies, this AFE sensitization platform could be combined with controlled NO_2_ or particulate exposures during early life to dissect how TRAP-induced oxidative stress and airway inflammation intersect with fungal allergen-driven Th2/Th9 and eosinophil pathways, thereby experimentally bridging mechanistic mouse data with epidemiologic observations on air pollution and childhood asthma. In this sense, the AFE model functions not only as an immunologic map of sensitization but also as a tractable screening system for pathway-selective interventions implemented before chronic airway remodeling becomes fixed.

Yours sincerely, 

Long Liang^△^^,^^✉^, Ying Luo^△^, Yuqiang Pei, Yongchang Sun^✉^

Department of Respiratory and Critical Care Medicine, Peking University Third Hospital,

 Beijing 100191, 

 China.

^△^These authors contributed equally to this work. 

^✉^Corresponding authors: Yongchang Sun, E-mail: suny@bjmu.edu.cn, ORCID: 0000-0003-3281-8854; Long Liang, E-mail: logan1733@163.com, ORCID: 0000-0002-1830-1907.

## Additional information

The online version contains supplementary material available at http://www.jbr-pub.org.cn/article/doi/10.7555/JBR.39.20250479.
