# Supplementary material for: Immune cell and cytokine profiles in a murine Aspergillus fumigatus extract sensitization model
Source: J Biomed Res. 2026 Sep 28;40(5):562–6. doi: 10.7555/JBR.39.20250479 (PMC13620457; doi:10.7555/JBR.39.20250479)
Supplement: Supplementary file 1 [file jbr-40-5-562-S1.pdf]

Available online at www.jbr-pub.org.cn

Open Access at PubMed Central

JBR

Journal of Biomedical Research, 2026 40(5): 1–4

Supplementary Data

Immune cell and cytokine profiles in a murine *Aspergillus fumigatus* extract sensitization model

Yongchang Sun△,✉, Ying Luo△, Yuqiang Pei, Long Liang✉

Department of Respiratory and Critical Care Medicine, Peking University Third Hospital, Beijing 100191, China.

△These authors contributed equally to this work.

✉Corresponding authors: Yongchang Sun, E-mail: suny@bjmu.edu.cn, ORCID: 0000-0003-3281-8854; Long Liang, E-mail: logan1733@163.com, ORCID: 0000-0002-1830-1907.

Received: 01 November 2025; Revised: 23 December 2025; Accepted: 30 December 2025; Available online: 06 January 2026; Published date: 28 September 2026

CLC number: R562.25, Document code: B

The authors reported no conflicts of interest.

This is an open access article under the Creative Commons Attribution (CC BY 4.0) license, which permits others to distribute, remix, adapt and build upon this work, for commercial use, provided the original work is properly cited.

© 2026 by Journal of Biomedical Research.

https://doi.org/10.7555/JBR.39.20250479

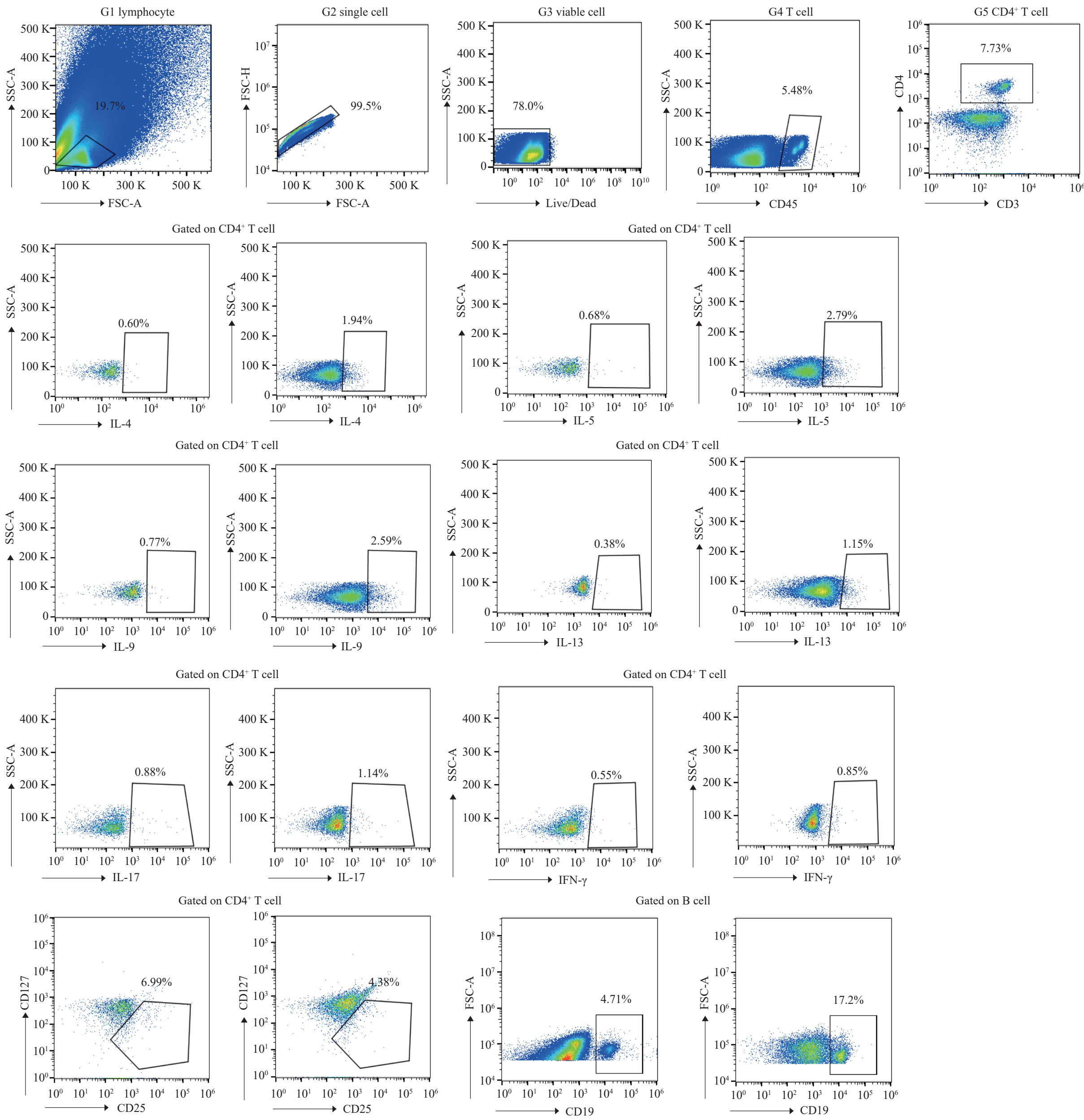

Supplementary Fig. 1 **CD4⁺ T cell subset alterations in the lung and blood of AFE-sensitized mice.** Flow cytometric analysis showing the gating strategy for CD4⁺ T cells expressing IL-4, IL-5, IL-9, IL-13, IL-17, and IFN-γ, as well as Treg cells and CD19⁺ B cells, in the lung and blood of AFE-sensitized mice. Abbreviations: AFE, *A. fumigatus* extract; Af, *Aspergillus fumigatus*; IFN-γ, interferon gamma; IL, interleukin; Treg, regulatory T cell.

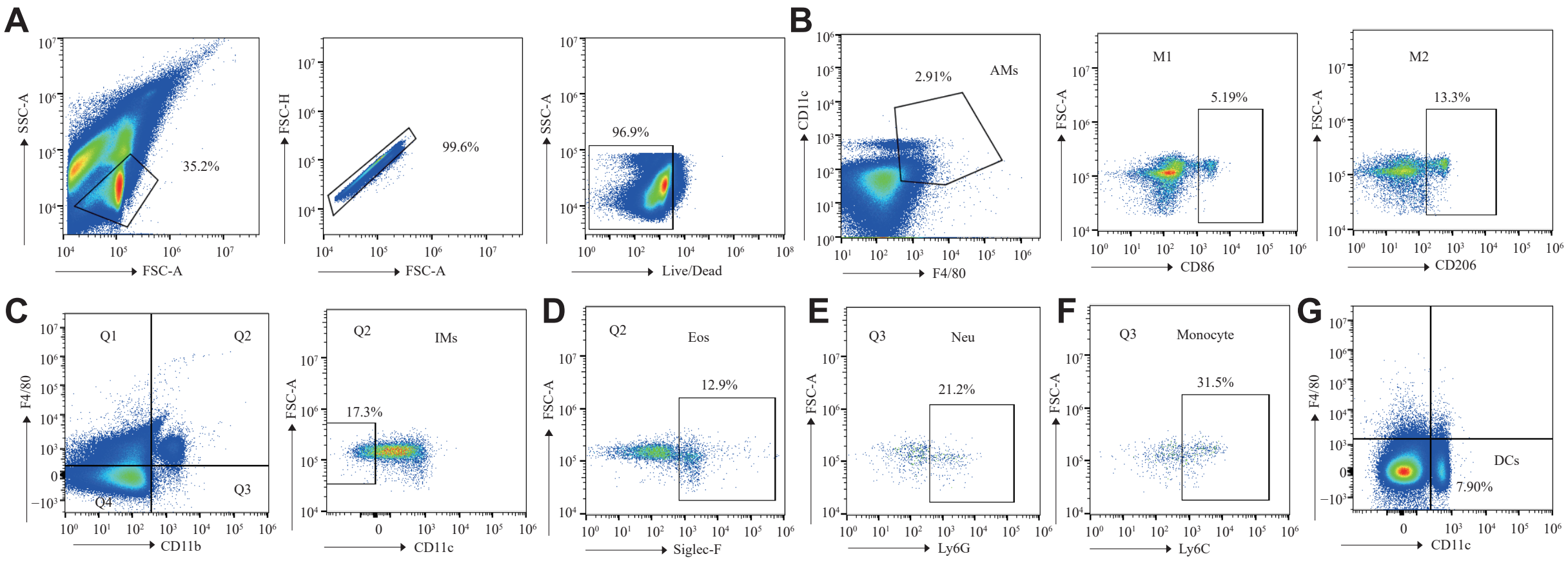

Supplementary Fig. 2 **Characterization of myeloid cell subsets in the lung and blood of AFE-sensitized mice.** A: Flow-cytometry gating scheme depicting population gating, singlet selection, and viability assessment. B: The gating of AMs and their subsets in the lung and blood. C: The gating of IMs in the lung. D: The gating of eosinophils in the lung and blood. E: The gating of neutrophils in the lung and blood. F: The gating of monocytes in the lung and blood. G: The gating of DCs in the lung and blood. Abbreviations: AMs, alveolar macrophages; DCs, dendritic cells; Eos, eosinophils; Neu, neutrophils; IMs, interstitial macrophages; M1/M2, M1-like/M2-like macrophages.

Supplementary Table 1 **Markers of various cells**

Cell types	Surface marker	Intracellular marker
Th2	$CD4^+$	$IL4^+/IL\text{-}5^+/IL\text{-}13^+$
Th1	$CD4^+$	$IFN\text{-}\gamma^+$
Th9	$CD4^+$	$IL9^+$
Th17	$CD4^+$	$IL\text{-}17^+$
Tregs	$CD4^+CD25^+CD127^–$	–
B cell	$CD19^+$	–
Alveolar macrophage	$F4/80^+CD11c^+$	–
Interstitial macrophage	$F4/80^+CD11b^+CD11c^–$	–
M1 macrophage	$CD86^+$	–
M2 macrophage	$CD206^+$	–
Macrophage (blood)	$F4/80^+CD11b^+Ly6C^+$	–
Eosinophil	$F4/80^+CD11b^+Siglec\text{-}F^+$	–
Neutrophil	$F4/80^-CD11b^+Ly6G^+$	–
Monocyte	$F4/80^-CD11b^+Ly6C^+$	–
Dendritic cells	$F4/80^-CD11c^+$	–

Supplementary Table 2 **Detailed information of the antibodies used in the study**

Reagents	Cat. No.	Concentration
Zombie UV™ Fixable Viability Kit	423107	1 : 200
APC/Cyanine7 anti-mouse CD45	103115	1 : 50
FITC anti-mouse CD4	100405	1 : 50
Brilliant Violet 785™ anti-mouse CD3	100231	1 : 50
Brilliant Violet 605™ anti-mouse IL-4	504125	1 : 50
APC anti-mouse/human IL-5	504305	1 : 50
Brilliant Violet 421™ anti-mouse IL-9	514109	1 : 50

Supplementary Table 2 **Detailed information of the antibodies used in the study** (continued)

Antibody name	Cat. No.	Concentration
PE anti-mouse IL-13	159403	1 : 50
PE anti-mouse IFN-γ	163503	1 : 50
APC anti-mouse IL-17A	506915	1 : 50
PE anti-mouse CD25	113703	1 : 50
APC anti-mouse CD127 (IL-7Rα)	158205	1 : 50
FITC anti-mouse CD86	159219	1 : 50
PE/Cyanine7 anti-mouse CD206 (MMR)	141719	1 : 50
PE anti-mouse F4/80	111603	1 : 50
Spark Red™ 718 anti-mouse CD11c	117371	1 : 50
PE/Fire™ 810 anti-mouse/human CD11b	101285	1 : 50
APC anti-mouse CD170 (Siglec-F)	155507	1 : 50
PE/Dazzle™ 594 anti-mouse Ly-6G	127647	1 : 50
Pacific Blue™ anti-mouse Ly-6C	128013	1 : 50
PE anti-mouse CD19	152407	1 : 50

All antibodies were purchased from BioLegend (San Diego, CA, USA).

Supplementary Table 3 **The gene primer sequences**

Genes (mouse)	Primer direction	Primer sequences (5′–3′)
Ifng	Forward	TGGCTGTTTCTGGCTGTTAC
	Reverse	TCCACATCTATGCCACTTGAGTT
Il1b	Forward	TGGCAGCTACCTGTGTCTTTC
	Reverse	GGATGGGCTCTTCTTCAAAGATG
Tnf	Forward	GATCGGTCCCCAAAGGGATG
	Reverse	CCACTTGGTGGTTTGTGAGTG
Il18	Forward	TGGTTCCATGCTTTCTGGACTCCT
	Reverse	TTCCTGGGCCAAGAGGAAGTGATT
Il4	Forward	CTCACAGCAACGAAGAACACC
	Reverse	CTGCAGCTCCATGAGAACACT
Il5	Forward	AAACTGTCCGTGGGGGTACT
	Reverse	CCTCGCCACACTTCTCTTTT
Il13	Forward	CCTGGCTCTTGCTTGCCTT
	Reverse	GGTCTTGTGTGATGTTGCTCA
Ccl17	Forward	CAGGAAGTTGGTGAGCTGGT
	Reverse	GACAGTCAGAAACACGATGGC
Il9	Forward	GAACATCACGTGTCCGTCCT
	Reverse	CGGCTTTTCTGCCTTTGCAT
Il22	Forward	GCTCAGCTCCTGTCACATCA
	Reverse	CAGTTCCCCAATCGCCTTGA
Gapdh	Forward	ACCCTTAAGAGGGATGCTGC
	Reverse	CCCAATACGGCCAAATCCGT
